# Cortical Microcircuit Mechanisms of Mismatch Negativity and Its Underlying Subcomponents

**DOI:** 10.3389/fncir.2020.00013

**Published:** 2020-03-31

**Authors:** Jordan M. Ross, Jordan P. Hamm

**Affiliations:** ^1^Neuroscience Institute, Georgia State University, Atlanta, GA, United States; ^2^Center for Behavioral Neuroscience, Georgia State University, Atlanta, GA, United States; ^3^Center for Neuroinflammation and Cardiometabolic Diseases, Georgia State University, Atlanta, GA, United States

**Keywords:** adaptation, deviance detection, interneuron, NMDA, somatostatin, parvalbumin, cortex, schizophrenia

## Abstract

In the neocortex, neuronal processing of sensory events is significantly influenced by context. For instance, responses in sensory cortices are suppressed to repetitive or redundant stimuli, a phenomenon termed “stimulus-specific adaptation” (SSA). However, in a context in which that same stimulus is novel, or deviates from expectations, neuronal responses are augmented. This augmentation is termed “deviance detection” (DD). This contextual modulation of neural responses is fundamental for how the brain efficiently processes the sensory world to guide immediate and future behaviors. Notably, context modulation is deficient in some neuropsychiatric disorders such as schizophrenia (SZ), as quantified by reduced “mismatch negativity” (MMN), an electroencephalography waveform reflecting a combination of SSA and DD in sensory cortex. Although the role of NMDA-receptor function and other neuromodulatory systems on MMN is established, the precise microcircuit mechanisms of MMN and its underlying components, SSA and DD, remain unknown. When coupled with animal models, the development of powerful precision neurotechnologies over the past decade carries significant promise for making new progress into understanding the neurobiology of MMN with previously unreachable spatial resolution. Currently, rodent models represent the best tool for mechanistic study due to the vast genetic tools available. While quantifying human-like MMN waveforms in rodents is not straightforward, the “oddball” paradigms used to study it in humans and its underlying subcomponents (SSA/DD) are highly translatable across species. Here we summarize efforts published so far, with a focus on cortically measured SSA and DD in animals to maintain relevance to the classically measured MMN, which has cortical origins. While mechanistic studies that measure and contrast both components are sparse, we synthesize a potential set of microcircuit mechanisms from the existing rodent, primate, and human literature. While MMN and its subcomponents likely reflect several mechanisms across multiple brain regions, understanding fundamental microcircuit mechanisms is an important step to understand MMN as a whole. We hypothesize that SSA reflects adaptations occurring at synapses along the sensory-thalamocortical pathways, while DD depends on both SSA inherited from afferent inputs and resulting disinhibition of non-adapted neurons arising from the distinct physiology and wiring properties of local interneuronal subpopulations and NMDA-receptor function.

## Introduction

Organisms are continuously inundated with sensory information. Given that the majority of incoming information is redundant or behaviorally unimportant, organisms need to be able to suppress the neural processing of irrelevant stimuli to conserve resources. Such adjustments are made possible by a process referred to as stimulus-specific adaptation (SSA; Adrian, 1926a,b; Adrian and Zotterman, 1926a,b; Nomoto et al., 1964; Pérez-González and Malmierca, 2014). Neurons can adapt to repetitive sensory information, reducing their firing responses at various stages of processing, from initial sensation to higher-order encoding, a phenomenon that occurs across all sensory modalities (McLaughlin and Kelly, 1993; Dalton, 2000; Wagner et al., 2006; Kohn, 2007; Pérez-González and Malmierca, 2014; Heil and Peterson, 2015). However, the sensory world is dynamic and the ability to perceive changes and adjust behavior accordingly determines the success of the organism. While organisms must be able to undergo SSA, they must also be able to quickly detect changes that differ from what has recently been experienced (or what can thereby be predicted), as it may signal salient information. This ability of neurons and neural systems to detect abrupt, unexpected variation from the constant, expected sensory milieu (or a set of presented stimuli) is known as deviance detection (DD) and typically involves an increase in firing responses (Malmierca et al., 2009; Antunes et al., 2010; Hamm and Yuste, 2016; Musall et al., 2017; Parras et al., 2017; Polterovich et al., 2018).

**Figure 1:**
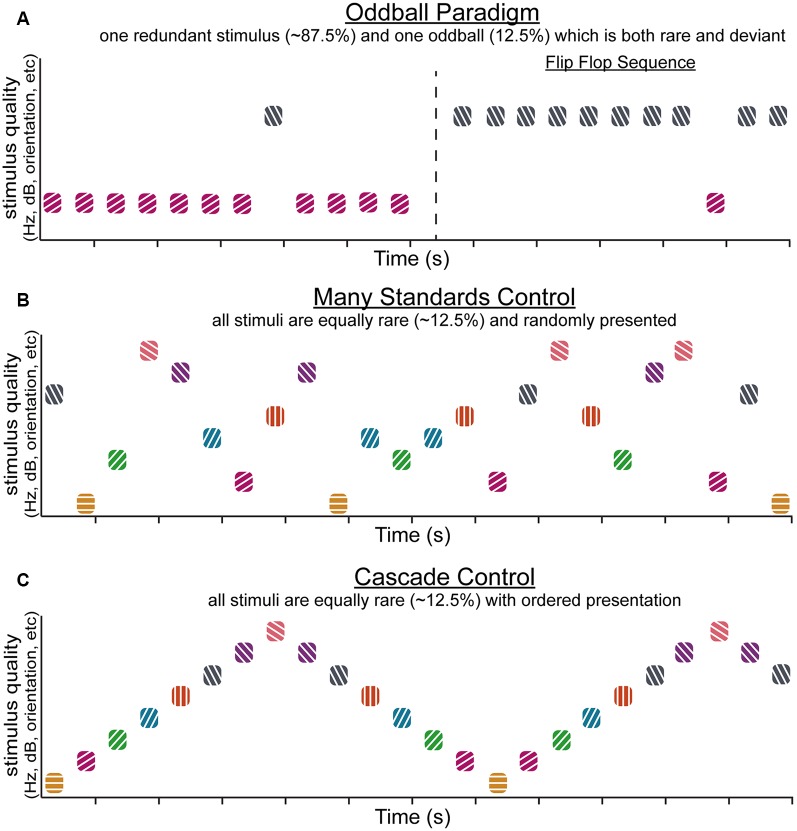
The oddball paradigm and commonly associated control sequences. Simplified schematics demonstrating various stimulus sequences that can be used to investigate stimulus-specific adaptation (SSA) and deviance detection (DD). The typical oddball sequence (**A**, left) utilizes two stimuli that differ in stimulus quality. One of the stimuli is designated the “redundant” and accounts for the majority of the presentations (in this case ~90%). The overabundance of redundant presentations establishes a regular pattern that is violated by “oddball” (or “deviant”) stimuli, which rarely occur (in this case ~10% of presentations). An extension of the oddball paradigm, the flip flop sequence (**A**, right) flips the redundant and oddball stimuli to control for differences in neural responses that might arise due to the physical characteristics of the stimuli. The many standards control sequence **(B)** presents several stimuli within a sensory modality that differ in terms of stimulus quality so that each appears with equally rare probability, in this case, ~12.5% of the time. There is no established pattern of stimulus presentations. The stimuli in the many standards sequence are presented randomly, unlike stimuli in a cascade control sequence **(C)**, where several stimuli appear with equally rare probability (again, in this case, ~12.5%) but are presented in ascending or descending order such that the difference in stimulus quality is the same for each presentation.

Together, SSA and DD represent important, complementary phenomena—the ability to adapt to contextually redundant information (SSA) while maintaining the ability to detect when a change occurs (DD) that might signal relevant, important information. Notably, most of the research regarding these phenomena has been carried out in the auditory and visual systems, and thoughtful experimental design efforts to differentiate SSA and DD (Figure 1) are an emerging trend in the study of sensory context processing (Harms et al., 2014; Chen et al., 2015; Hamm and Yuste, 2016; Wiens et al., 2019). For example, sensory “oddball” paradigms involve the presentation of a repetitive, highly probable “standard” (or “redundant”) stimulus typically occurring between 75–95% of trials (which occur rapidly, at least once every second) with a rarer interspersed “target” (or “deviant”) stimulus (Figure 1A). Studies employing a single “oddball” paradigm are the most common, yet alone they cannot differentiate SSA and DD. A simple difference wave (in the EEG) or difference in neuronal spike rate between “redundants” and “deviants” conflate these two components. Control paradigms such as “flip flop” sequences (Figure 1A; i.e., two back to back oddball runs where the deviant and the redundant are swapped) ensure DD is not a function of physical stimulus characteristics. Another commonly used technique, the “many standards” control (Figure 1B) allows researchers to determine whether DD signatures are due to the relative rarity of “deviant” stimuli or result from true detection of deviations from expected patterns (Schröger and Wolff, 1996; Jacobsen and Schröger, 2001, 2003; Jacobsen et al., 2003b). Further, “cascade” sequences (Figure 1C) are used to ensure that the ordering effects in basic “oddball” presentations do not contribute to apparent DD. Stimuli in the cascade control are always preceded by the same stimulus, like the typical oddball sequence, and unlike the many-standards control. Also unlike the many-standards control, the cascade sequence establishes a pattern of stimulus presentations with (overall) less influence of SSA.

**Figure 2:**
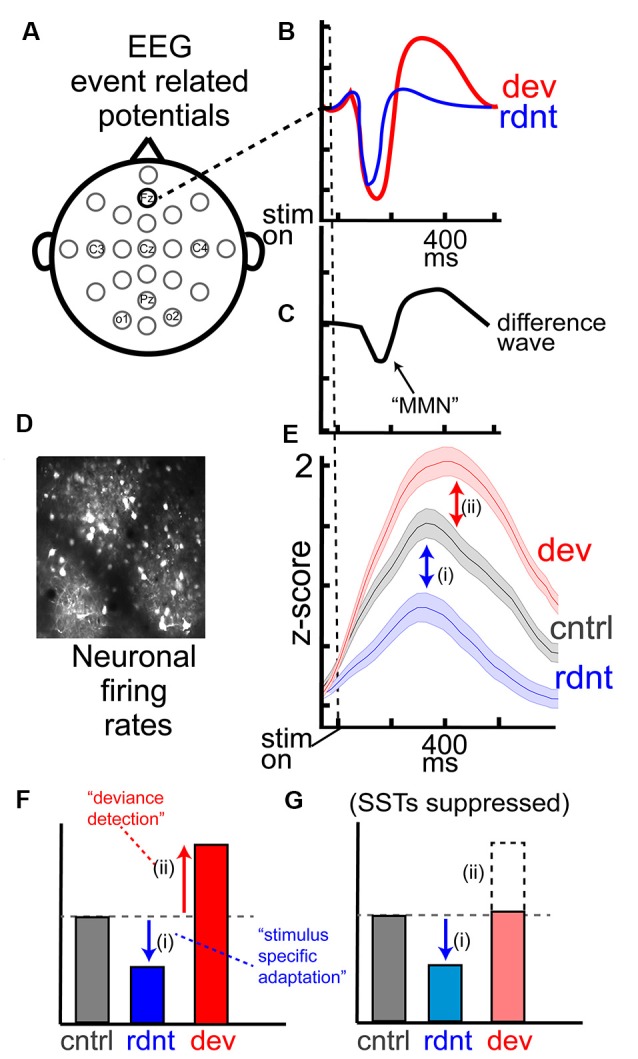
EEG measurements of mismatch negativity (MMN) and key components at the level of cortical neurons. **(A)** Typical EEG layout, with **(B)** event-related potentials (ERPs) from the frontal sensor (Fz; often used for auditory MMN) averaged over trials for the redundant and deviant stimuli. **(C)** MMN is commonly computed as the first peak difference wave between the waveforms in (**B**; waveforms reflect theoretical data from hundreds of studies). **(D)** Direct records of neuronal activity with two-photon calcium imaging in awake mouse layer 2/3 of sensory cortex reveal **(E)** suppressed responses to the redundant stimulus [SSA; (i)] and augmented responses to a deviant stimulus [DD; (ii)] compared to the same stimulus presented during the many-standards control. **(F,G)** Theoretical barplot from effects in **(E)** depicts that DD, but not SSA, are influence by chemicogenetic suppression of SSTs in the visual cortex (data in **D–G** adapted from Hamm and Yuste, 2016).

While SSA and DD are typically measured at the neuronal level, they likely reflect circuit-level computations (Natan et al., 2015, 2017; Hamm and Yuste, 2016), and are robust when measured with gross-electrophysiological techniques reflecting summed activity within a neocortical region (local field potential, LFP; electroencephalogram, EEG). Thus, SSA and DD can be assessed at multiple levels in multiple species, including humans where non-invasive measurements of neurophysiology remain constrained to a more gross, macro-level (i.e., EEG or MEG). Despite their presence across species, human EEG studies, especially in clinical and neuropsychiatric research, have focused instead on an aggregate measure of context processing: the mismatch negativity (MMN; Näätänen, 1995; Näätänen and Alho, 1995b; Tiitinen et al., 1997). MMN is an event-related potential (ERP) wherein a more negative scalp potential (occurring about 150 ms post-stimulus onset) is elicited by the “deviant” stimulus than by the “redundant” stimulus in an oddball paradigm (Figures 2A–C). Diminished or absent MMN is a classic, highly replicated biomarker for sensory context processing deficits common in schizophrenia (SZ) and other psychotic disorders (Näätänen et al., 2011, 2014; Lavoie et al., 2019; Tada et al., 2019), so efforts to describe the biological substrates and mechanisms of human MMN remain paramount. Indeed, some progress has been made to understand MMN generation (Garrido et al., 2009), but confoundingly, MMN comprises both SSA and DD (Figure 2E). These subcomponents are rarely assessed separately in human clinical studies (which often just involve a single “oddball” paradigm), and if these subcomponents depend on different neurobiology or circuit functions, a direct interpretation of the neural underpinnings of SZ-MMN deficits will remain challenging.

**Figure 3:**
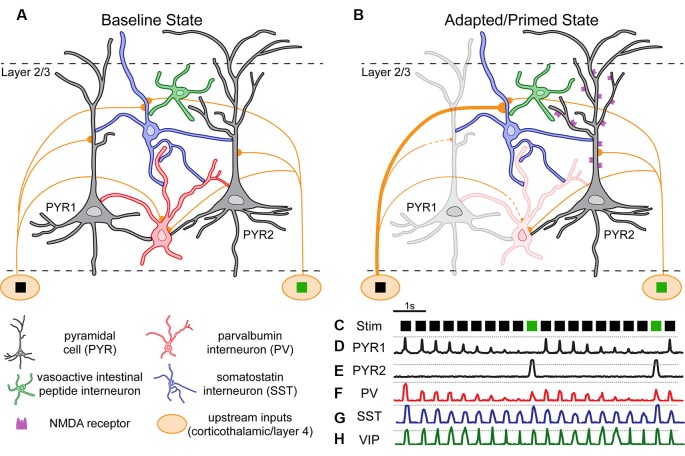
Hypothetical model of layer 2/3 cortical circuitry and neuronal responses during an oddball paradigm. In a theoretical “baseline” (unadapted) state **(A)**, circuits of pyramidal and interneuron subtypes in-display interconnections based on tracing and electrophysiology studies in the mouse cortex. Cells receive inputs from thalamus or upstream layer 4 neurons selective for one of two stimuli (e.g., black square, green square). Pyramidal cells (PYR1 and PYR2) show stimulus selectivity. **(B)** In an adapted/primed state some inputs/cells have increased (thickened) or decreased (lightened) excitability [**(C)** i.e., during a classic “oddball” paradigm]. **(D–H)** Theoretical activity traces from cells in **(A,B)**.

This review aims to examine recent studies regarding the mechanisms of SSA and DD in animals and, when appropriate, compare them to human studies of MMN. Unfortunately, it remains unclear whether human SZ populations exhibit deficits in SSA, DD, both, neither, and/or some additional MMN-relevant component, yet the fact that DD, in particular, may depend on some neuronal functions with known SZ relevance (e.g., interneurons) merits consideration (Hamm and Yuste, 2016). Here we show that the emerging literature demonstrates that, indeed, SSA and DD can be separated experimentally at the cellular and circuit level in rodents, but it remains unclear how they are related mechanistically and concerning the underlying cells and circuits which generate them. Therefore, we aim to discuss the underlying circuits identified through animal experimentation, proposing a hypothetical model of these circuits in layer 2/3 of the neocortex (Figure 3). Our model, though limited by virtue (Box et al., 2005), will hopefully prove useful for future investigations of sensory context processing in the neocortex.

## The “Oddball” Paradigm

To quantify sensory context processing in human participant samples, researchers often record EEG and employ an “oddball” paradigm (Figures 1, 2A–C). This paradigm and related studies have been thoroughly reviewed elsewhere (Picton, 1992; Näätänen, 1995), and the basic structure of stimulus presentation is described above (Figure 1). Importantly, these stimuli can be virtually of any sensory modality, all of which have produced MMN analogs in the EEG (Tiitinen et al., 1997; Shinozaki et al., 1998; Pause and Krauel, 2000; Rosburg et al., 2007; Escera et al., 2014; Kremlacek et al., 2016). One of the strengths of the MMN is that it is independent and unaffected by active attention to the presented stimuli (Schröger et al., 1992; Näätänen and Alho, 1995a; Marshall et al., 1996; Tiitinen et al., 1997; Fischer et al., 2000; Näätänen, 2000; Ibáñez et al., 2009; Näätänen et al., 2010), unlike, for example, the P300, which requires an ongoing target detection task, occurs 150 ms later than the MMN, and likely involves synchronized activity across many sensory and non-sensory cortical and subcortical brain areas. This added complexity makes P300 less valuable for a pure assessment of contextual modulation of sensory processing (Picton, 1992; Näätänen, 1995; Ethridge et al., 2015). This is especially important given that people with SZ often have both attentional and pre-attentive processing deficits (Rabinowicz et al., 2000; Schechter et al., 2006; Elshaikh et al., 2015; Hamm et al., 2015; Javitt and Freedman, 2015), and, thus, a marker of pre-attentive context processing like the MMN is advantageous for animal experimentation where attention may be difficult to obtain or gauge.

In general, the fact that “deviant” stimuli in the “oddball” paradigm elicit an enhanced response in sensory cortex matches well with results obtained across multiple sensory modalities and from several species (Featherstone et al., 2018; Tada et al., 2019) including non-human primates (Javitt et al., 1992; Ueno et al., 2008; Komatsu et al., 2015), rats (Nakamura et al., 2011; Shiramatsu et al., 2013; Harms et al., 2014), and mice (Umbricht et al., 2005; Ehrlichman et al., 2008; Chen et al., 2015), suggesting that the oddball paradigm is well suited for studying contextual modulation of sensory cortical responses in both human and animal studies and that underlying mechanisms are likely conserved across sensory modality. Still, establishing a one-to-one correspondence of gross-level brain potentials/waves (such as the N1, P2, and MMN) between humans and animals (Ehrlichman et al., 2008), and even between sensory modality within human samples (Kremlacek et al., 2016), has not been straightforward. The latencies and shapes of ERPs within and across species depend on several factors not directly related to the fundamental sensory or cognitive “computation,” including the speed of neuronal transmission from primary sensory afferents to neocortex and the location of recording electrodes with regards to the generator of the ERP waveform (Luck and Kappenman, 2013).

On the other hand, animal studies do offer a direct measure of the firing responses of individual neurons within the sensory cortex, and enable deeper, more direct insight into biological mechanisms (Figures 2D,E). An additional advantage presented by animal models is that virtually all experimental practices employed in human studies have homologs for animal experimentation, allowing for direct comparison while simultaneously offering more mechanistic insight. For instance, ketamine, along with another N-methyl-D-aspartate receptor (NMDAR) antagonists, is known to reduce the MMN in humans as well as rodents and non-human primates (Ehrlichman et al., 2008; Gil-Da-Costa et al., 2013; Chen et al., 2015; Haaf et al., 2018; Schuelert et al., 2018).

Furthermore, the oddball task applies to a wide variety of experimental assays in animal research. In addition to the standard scalp and intracranial LFP recordings (Ayala et al., 2012; Ayala and Malmierca, 2015) that are relatable to human EEG measures, mouse models, in particular, are compatible with more cutting-edge methods that allow direct visualization and/or manipulation of neural activity, such as electrophysiological cell recordings (Taaseh et al., 2011; Chen et al., 2015; Duque et al., 2016; Parras et al., 2017), two-photon (2P) imaging, and Opto/chemico-genetics (Natan et al., 2015; Hamm and Yuste, 2016). These newer imaging techniques provide significantly improved spatial resolution over traditional methods. For example, recent work using a combination of two-photon microscopy and LFP recordings verified the presence of an MMN-like LFP response and established that both SSA and DD could be reliably measured at the level of individual neurons (Hamm and Yuste, 2016).

Mice also allow for genetic dissection of specific cell populations. This represents a significant improvement over traditional methods that were only able to measure from a heterogeneous population with limited spatial resolution. Recent studies have focused on principal neuron populations as well as inhibitory interneurons and are beginning to yield significant insights into the mechanisms of MMN and sensory context processing (Natan et al., 2015; Duque et al., 2016; Hamm and Yuste, 2016; Musall et al., 2017). Additionally, the oddball paradigm has been used in conjunction with pharmacology (Ehrlichman et al., 2008, 2009; Bristow et al., 2016; Aleksandrov et al., 2018; Harms et al., 2018; Lee et al., 2018), optogenetics (Natan et al., 2015, 2017), and chemogenetics (Hamm and Yuste, 2016) to isolate mechanisms responsible for the components of MMN. The genetic access along with the available manipulations that animal models afford allows significant insights to the mechanisms of MMN in human populations, both basic and clinical.

Yet, because the correspondence of mouse responses to human-like MMN potentials measured at the scalp is unclear, animal studies of MMN-like processing have been typically held to the additional criterion of differentiating true DD from SSA (Harms et al., 2016). As described above, this requires at least two additional stimulus presentation paradigms, the flip flop and the many-standards paradigm (Figure 1; Hamm and Yuste, 2016), enabling the researcher to separate DD in a neuron’s augmented response to the “oddball” from its simple preference to that stimulus and the absence of SSA (Harms et al., 2016). Indeed, mouse sensory cortices do display both SSA (i.e., response to stimulus in many-standards control is greater than the response to the same stimulus when presented repetitively in oddball) and DD (response to stimulus in oddball is greater than the response to the same stimulus in many-standards control), and the ability to apply the above-mentioned technologies is leading to important inferences about the mechanisms of SSA and DD (Hamm and Yuste, 2016; Musall et al., 2017; Parras et al., 2017; Polterovich et al., 2018), which we will describe below. It remains unknown whether SSA and DD are distinct processes and whether they are dependent on one another—in other words, must a neuronal circuit undergo adaptation to detect deviations from previous stimulation, and vice versa, is DD necessary for maintaining stimulus adaptation?

## Specific Stimulus Adaptation

Adaptation or reduced neuronal responsivity, selective to repeated stimuli occurs in all primary sensory cortices (Figures 2D–F) but is also measurable in some subcortical structures (Nelken, 2014). For example, in auditory processing SSA can be detected in the inferior colliculus, a midbrain structure involved in auditory processing that lies upstream of both primary thalamic nuclei and auditory cortex. GABA, primarily through GABAa receptors, appears to exert its effects through overall gain-control in the inferior colliculus, by regulating the magnitude of neural excitation to repeated stimulation (Duque et al., 2014). The local inhibition accounts for a significant proportion of SSA but cannot completely explain the altered neural responses (Ayala and Malmierca, 2018), indicating other mechanisms play a role as well. Similarly, cholinergic and endocannabinoid systems act to modulate SSA subcortical auditory and olfactory responses *en masse* but are not responsible for generating SSA (Ayala and Malmierca, 2015; Valdés-Baizabal et al., 2017; Ogg et al., 2018). Iontophoretic application of both acetylcholine and cannabinoid agonists appear to increase responses (through muscarinic receptors and cannabinoid receptors type 1, respectively) specifically to repetitive stimulations, thereby reducing SSA, without affecting neural responses to the deviant tone (Ayala and Malmierca, 2015; Valdés-Baizabal et al., 2017). All together these effects likely propagate to downstream primary cortices, thus altering the input to these regions in a stimulus specific manner and driving behavioral responses. There is indirect evidence to suggest that this is the case as stimulation of cholinergic release in the olfactory bulb is sufficient to reinstate olfactory bulb responses to repetitive stimuli (in both anesthetized and awake conditions) and is sufficient to reinstate behavioral investigatory behaviors to repetitive olfactory stimuli (Ogg et al., 2018); however, it remains unclear the extent to which these processes directly contribute to SSA in primary sensory cortices to contribute to cortical SSA and perceptual adaptation.

At the level of individual neurons, SSA can occur at the input-output stage by changing the intrinsic response properties of presynaptic neurons, such as spike frequency (Fairhall et al., 2001; Wilent and Contreras, 2005; Pozzorini et al., 2013; Ogg et al., 2015), or by modulating the strength of input onto postsynaptic neurons *via* synaptic depression (Abbott et al., 1997; Tsodyks and Markram, 1997; Anwar et al., 2017; Musall et al., 2017). For example, across all sensory cortices, repetitive stimulation induces spike frequency adaptation, an increase in neuronal firing threshold following an initial response that reduces the firing frequency of the neuron. Spike frequency adaptation is long-lasting in neocortical pyramidal cells (PYRs) and can cause temporal decorrelation of output spikes (Pozzorini et al., 2013). Such mechanisms at the level of individual neurons or populations of neurons are putative mechanisms by which SSA occurs in the global cortical system. Single synapses can also change in response to repeated stimulation, demonstrating short-term facilitation, depression, or a combination of the two which acts to dynamically filter sensory input (Suzuki and Bekkers, 2006; Kuo and Trussell, 2011; Nikolaev et al., 2013). In essence, sensory input activates a specific population of neurons that are all tuned towards that specific input. Continual presentations of that stimulus ultimately lead to synaptic depression of those neurons, which in turn reduces the excitatory drive onto downstream cortical neurons (Mill et al., 2011). While this adaptation is measurable in single neurons, individual neurons participate in larger networks so changes in a population of neurons propagate throughout the sensory system, which is relevant to wide-scale sensory coding. It is important to remember that networks of neurons rather than individual neurons are responsible for sensory adaptation, even though adaptive processes of individual neurons likely contribute to cortical SSA, as SSA cannot be explained by intrinsic properties of single neurons alone (Mill et al., 2011; Solomon and Kohn, 2014; Malmierca et al., 2015).

Cortical SSA has also been measured in the absence of cortico-cortical synaptic depression, instead finding that repeated sensory stimulation reduces thalamocortical input (Chung et al., 2002). This suggests that decreased thalamic input to cortical regions contributes to cortical SSA during repeated stimulation, such as during an oddball paradigm (Natan et al., 2015). Thalamocortical synaptic depression was once thought to exist only during development but evidence demonstrates it is conserved in adults (Blundon et al., 2011), making it an attractive candidate for an underlying mechanism of cortical SSA. Given that afferent projections from thalamus represent the primary pathway for most sensory information to sensory cortical regions, altered thalamic input to the cortex, or thalamocortical gating, likely plays an important role in controlling cortical responses to sensory input (Hillenbrand and van Hemmen, 2002; Wang et al., 2010; Whitmire et al., 2016). While nearly all sensory information is relayed through the thalamus to sensory cortices, olfactory information bypass the thalamus entirely but olfactory cortices still exhibit SSA (McCollum et al., 1991; Wilson, 1998a,b; 2000; Best and Wilson, 2004), suggesting that SSA is a combination of cortical synaptic depression as well as thalamocortical gating (Lundstrom et al., 2010; Blundon and Zakharenko, 2013; Nelken, 2014).

While the role of SSA specifically in SZ-related MMN deficits remains to be demonstrated (Michie et al., 2016), people with SZ can manifest what appears to be reduced cortical adaptation (Javitt and Freedman, 2015; Javitt and Sweet, 2015; Andrade et al., 2016) and thalamocortical gating (Adler et al., 1998). Thalamocortical gating alone cannot explain SSA (Lundstrom et al., 2010; Nelken, 2014), and ERP studies in general in SZ, including the “P50 gating” ERP, often cannot disentangle true deficits in ERP adaptation from simply reduced baseline ERPs (i.e., reduced cortical response to the initial stimulus vs. the repeated one (Clementz et al., 1998; Hamm et al., 2014). Still, the evidence linking SZ to altered thalamocortical connectivity extends beyond MMN and P50 paradigms and this disconnect is thought to underlie several aspects of symptomology (Sodhi et al., 2011; Klingner et al., 2014; Chen et al., 2019; Hua et al., 2019; Huang et al., 2019; Tu et al., 2019). It is noteworthy that some genetic SZ animal models possess altered thalamocortical networks (Chun et al., 2014; Kröcher et al., 2015), and administering ketamine, which blocks NMDARs to produce SZ-like phenotypes in healthy patients and animals alike, alters thalamocortical connectivity (Höflich et al., 2015; Becker et al., 2016; Furth et al., 2017). If reduced SSA does exist in SZ, it would remain difficult to determine the extent to which this is related to reduced thalamocortical connectivity, since reduced signaling would lead to smaller baseline responses to stimuli in sensory cortex (which has been demonstrated in SZ; Rosburg et al., 2008; Hamm et al., 2014). Reduced drive, to begin with, would effectively appear to reduce adaption when normalized (Clementz et al., 1998; Patterson et al., 2008). Additionally, from a cognitive perspective encoding “redundancy” or sensory context would be weaker when the signals are weaker in general.

One point to consider in this problem is that NMDAR blockade, a face-valid model of SZ sensory processing features, also reduces MMN in humans and animals (Ehrlichman et al., 2008; Gil-Da-Costa et al., 2013; Haaf et al., 2018; Schuelert et al., 2018). The nature of how NMDAR blockade affects the time-course and oscillatory aspects of auditory MMN has led to the interpretation that DD specifically is NMDAR-dependent (Javitt et al., 1996; Lee et al., 2018). There remains some discrepancy regarding whether and how NMDARs are involved in SSA (Farley et al., 2010; Chen et al., 2015). Farley et al. (2010) recorded multiunit activity in the auditory cortex of anesthetized rats and showed that systemic NMDAR blockade with MK801 did not affect gross SSA. On the other hand, Chen et al. (2015) report a significant effect of direct MK801 infusion on SSA in excitatory neurons based on whole-cell recordings in the auditory cortex of anesthetized mice. Interestingly, this effect effectively eliminated all stimulus-driven firing responses, so it remains unclear whether some aspects of SSA may have survived in excitatory neurons with lower doses of NMDAR blockade and/or in awake preparations, perhaps inherited from upstream sources. Additional work will be needed involving local NMDAR block at different concentrations.

It is important to note that sensory adaptation can be broadly defined as any stimulus- or context-dependent modulation of sensation or perception, and is a phenomenon that has been described across all modalities and all stages of sensory encoding and processing. One such form of sensory adaptation is forward suppression, in which processing of a stimulus can be modulated by a different immediately preceding stimulus (Plomp, 1964; Relkin and Turner, 1988; Scholes et al., 2011). Work in the auditory system has isolated two separate mechanisms that produce suppressed cortical responses on different timescales. Forward suppression in auditory cortex can last for hundreds of milliseconds; however, suppression in the first 100 ms seems to be a result of GABAergic postsynaptic inhibition whereas suppression beyond the first 100 ms is mediated by synaptic depression at thalamocortical projections due to a switch from burst firing to single action potential firing (Calford and Semple, 1995; Brosch and Schreiner, 1997; Wehr and Zador, 2005; Bayazitov et al., 2013). In this review, we refer to SSA, which can be thought of as a stimulus-specific, repetition-dependent form of forward suppression. The bursting switch which underlies auditory cortex forward suppression is also thought to explain part, but not all, of SSA (Bayazitov et al., 2013); however, this has not been systematically tested. Forward suppression as well as other forms of sensory adaptation, such as sensory gating, may be embedded in SSA and the oddball paradigm (Boutros et al., 1995; Wang et al., 2010); however, they likely affect all stimuli (redundant, deviant, and/or control stimuli) equally.

SSA can also operate on different timescales, with cortical responses exhibiting reduced responses to experienced stimuli for milliseconds to days (Condon and Weinberger, 1991; Ulanovsky et al., 2004; Kato et al., 2015). As with forward suppression, the different timescales of SSA are thought to rely on separate mechanisms. For example, long-lasting multiday adaptation appears to reflect increased recruitment of inhibitory interneurons, which can be reversed if the stimulus becomes behaviorally relevant (Kato et al., 2015). Therefore, the timing of both stimulus presentation and length of experimental paradigms represent important methodological considerations for further dissecting mechanisms of SSA.

## Deviance Detection

Like SSA, DD occurs in primary cortices (Figures 2E,F; Hamm and Yuste, 2016; Musall et al., 2017; Parras et al., 2017; Polterovich et al., 2018) as well as sub-cortical structures (Kohn, 2007; Anderson et al., 2009; Malmierca et al., 2009; Antunes et al., 2010) and measurable DD is seemingly stronger at subsequent downstream processing stages (Parras et al., 2017). Notably, in auditory paradigms brainstem nuclei do not appear to contribute to SSA or DD; however, subcortical neurons exhibiting SSA and DD receive strong input from the primary auditory cortex (Ayala et al., 2015). Therefore, it was previously postulated that DD measured in sub-cortical structures was a reflection of DD in primary sensory cortices backpropagating to lower regions (Nelken and Ulanovsky, 2007). However, deactivating sensory cortices does not appear to affect subcortical DD (Antunes and Malmierca, 2011; Anderson and Malmierca, 2013; Malmierca et al., 2015), suggesting DD may be independently generated or enhanced at each stage (Ayala and Malmierca, 2012). Recent work in the somatosensory cortex indicates cortical DD is due primarily to intracortical circuitry at specific cortical layers that may be enhanced by input from subcortical structures (Musall et al., 2017). In the visual cortex, DD may not be present in initial thalamic inputs at all, originating within intracortical circuits entirely (Hamm et al., 2018).

Intracortical circuits comprise complex excitatory-inhibitory interactions to shape sensory processing (Wood et al., 2017). The activity of excitatory neurons is sculpted by feedback from inhibitory interneurons, which makes them an interesting focus for understanding information processing within the circuit. Two of the largest populations of GABAergic inhibitory interneurons, parvalbumin- (PV) and somatostatin- (SST) positive interneurons, have gained significant attention in studies of DD, especially in the auditory cortex where they are known to influence representations of auditory stimuli. Interestingly, optogenetic stimulation of PVs in the auditory cortex increases the functional connectivity of the thalamocortical circuit (Hamilton et al., 2013), which may enhance processing, and even perception, of sensory inputs. In support of this, stimulation of PVs in the auditory cortex has been shown to enhance behavioral performance on tone frequency detection tasks, while suppression decreases behavioral auditory discrimination (Aizenberg et al., 2015). However, optogenetic stimulation of both PVs and SSTs in the auditory cortex can cause contradictory and confounding results when viewing the single-unit activity of aspects of auditory processing (Seybold et al., 2015; Phillips and Hasenstaub, 2016). Future work should include careful design and interpretation of causal manipulations of interneuron populations to dissect how interneurons function (both in isolation and in concert) to control cortical functions and sensory processing. Besides, other classes and sub-classes of neocortical inhibitory neurons, such as vasoactive intestinal polypeptide (VIP) neurons which often inhibit other inhibitory interneurons, have yet to be studied in this context and future studies should aim to include this unique and comparatively understudied class of interneurons.

Beyond understanding how cortical interneuron populations contribute to basic sensory processing, it is of interest to understand how they contribute to SSA and DD specifically. The use of transgenic animal models (Feil et al., 2009) allows for specific investigation and manipulation of subpopulations of neurons during the oddball paradigm to characterize neural activity. For example, studies using electrophysiological recordings of excitatory PYRs as well as PV and SST interneurons in auditory cortex supports evidence that each of these cell types demonstrate oddball driven responses (Chen et al., 2015; Natan et al., 2015). That is, these significant effects were computed between responses to deviants vs. redundants, potentially involving both SSA and DD. While Chen et al. (2015) included a separate analysis of genuine DD (i.e., responses to deviants vs. a many-standards control), PYRs, SSTs, and PVs all lacked significant DD in their spiking output (an effect potentially influenced by anesthesia). PYRs nevertheless demonstrated both early (0–100 ms after tone onset) and late (200–400 ms after tone onset) phase oddball effects (Chen et al., 2015), which align with the late component signals detected in human MMN and supports the use of oddball paradigms in mouse models as translationally applicable to humans. Other studies using mouse models have also demonstrated neuronal predictive activity that gives rise to large mismatch responses when expected patterns are violated, which mimics human MMN responses (Parras et al., 2017). Characterizing and understanding the contribution of interneuron activity during oddball paradigms may reveal physiological mechanisms of SSA, DD, and composite MMN that are relevant to understanding these phenomena in humans.

The fact that interneurons show oddball and other prediction error-driven activity (Chen et al., 2015; Garrett et al., 2020), suggests GABAergic interneurons may play a role in modulating DD in PYRs, which putatively gives rise to a perception of novelty or deviance. By recording from excitatory PYRs while opto-/chemogenetically modulating PVs and SSTs, researchers have been able to dissect contributions of these interneurons to DD in excitatory neocortical cells. Optogentically silencing PVs in the auditory cortex results in loss of overall gain control, equally enhancing responses to repeated stimuli, thereby reducing SSA, and deviant stimuli. However, silencing SSTs enhanced the firing rate of excitatory cells only in response to repeated stimuli (i.e “redundants”), without altering the firing rate in response to deviant stimuli (Natan et al., 2015). While this auditory cortex study did not specifically dissect DD from SSA with control paradigms, another study did and found that chemogenetic suppression of SSTs in the visual cortex reduces DD in excitatory neurons (Figure 2G; Hamm and Yuste, 2016). While differences in the use of control paradigms preclude a direct comparison of these effects, it’s important to note that, in both studies, SSTs appear to impart context selective inhibition on PYRs (while PVs do not). Further, the apparent differences on which stimulus type SSTs appear to exert the largest effect (redundant vs. deviant) could arise from differences in experimental paradigm [inclusion of control paradigms; optogenetic (Natan et al., 2015) vs. chemogenetic (Hamm and Yuste, 2016) manipulation of SSTs; sensory cortex studied]. Methods of interneuron silencing are important to consider as optogenetic suppression offers temporally precise and highly transient inactivation of desired cells, while chemogenetic suppression lasts several minutes to hours. This methodological difference means that chemogenetic suppression may affect all aspects of the oddball paradigm, affecting the overall encoding of the context or even giving rise to an adaptive rebalancing of inhibition/excitation ratios, for instance, while optogenetic suppression can be induced at discrete phases of the paradigm, which could contribute to the differences reported here. Despite contradictory results, these studies suggest a conserved role of SST interneurons in the contextual processing of stimuli in V1 and A1 cortices. Interestingly, postmortem analysis of SZ brain tissue demonstrates reduced and aberrant SST activity in the neocortex (Hashimoto et al., 2008a,b; Fung et al., 2010, 2014; Volk and Lewis, 2013), which poses an exciting link between the role of SSTs in SSA and/or DD and deficient MMN in SZ patients.

## Two Sides of the Same Coin?

As mentioned above, MMN and the use of oddball paradigms without additional controls have resulted in composite studies of SSA and DD, with DD often being assumed to be the simple absence of SSA in neural populations. However, using standard and deviant auditory stimuli of the same frequency but different intensities or localization is still capable of provoking an MMN response in humans, demonstrating that DD occurs in adapted populations and is not simply the absence of SSA (Schröger and Wolff, 1996; Jacobsen et al., 2003a; Althen et al., 2011; Shestopalova et al., 2018). Therefore, more focus has been on determining whether DD truly reflects a violation of expected patterns or rarity of an event. If DD reflects rarity, the magnitude of the response to the deviant stimulus would be the same whether the stimulus was deviant or simply rare. Alternatively, if DD reflects violation of expectations, truly deviant stimuli, those that disrupt expected patterns, would elicit a larger response than rare stimuli. Teasing these hypotheses apart has been of recent focus in animal models, as numerous paradigmatic controls have been implemented (Jacobsen and Schröger, 2001, 2003; Jacobsen et al., 2003b; Harms et al., 2014; Harms, 2016).

In addition to paradigm controls, researchers are using cell- and circuit-based manipulations to separate SSA and DD (Strelnikov, 2007). For example, in the inferior colliculus cholinergic modulation appears to affect SSA to repetitive auditory stimuli without altering DD (Ayala and Malmierca, 2015). Cholinergic modulation has also been shown to affect the MMN in neurotypical humans (Caldenhove et al., 2017); however, whether this is due to basic stimulus processing or novelty detection requires further investigation. In contrast, DD, but seemingly not SSA, depends on signaling from SST interneurons, at least in the primary visual cortex, as inhibiting them abolishes DD while sparing SSA in principal neurons of primary visual cortex (Hamm and Yuste, 2016). Additionally, NMDA receptors may support oddball-driven responses and/or DD as blockade of NMDA receptors reduces MMN (Ehrlichman et al., 2008; Chen et al., 2015; Harms, 2016; Chien et al., 2019) but proper controls still need to be employed to determine if this reduction is due to altered DD or SSA (Harms, 2016). Again, there is conflicting evidence regarding the extent to which the NDMAR function contributes to SSA, making it difficult to discern its relationship to DD (Farley et al., 2010; Chen et al., 2015).

Taken together the evidence supports conclusions drawn from human MMN studies and suggests that DD is a complex process that is separable from SSA in neural recordings (Csépe, 1995; Ruusuvirta et al., 1998; Jung et al., 2013; Shiramatsu et al., 2013; Harms et al., 2014; Chen et al., 2015; Hamm and Yuste, 2016; Kum et al., 2019); however, additional work is required to truly dissect individual mechanisms of each process. It has yet to be demonstrated whether DD in the cortex can exist without the presence of a locally, alternatively “adapted” population of neurons (or at least adapted upstream inputs). We have combined findings across several human and animal studies to build a theoretical model of cortical MMN wherein DD and SSA are separable, but wherein DD would depend on SSA (but not the reverse).

The basic model of layer 2/3 is laid out in Figure 3. In a baseline state (Figure 3A), upstream inputs synapse onto inhibitory interneurons (PV/SST) and PYRs. During an oddball paradigm (Figure 3B), the neocortical circuit enters a state which is both adapted and primed for DD. Repetitive stimulation (i.e., the same stimulus; Figure 3C) results in synaptic depression of inputs from upstream neurons selective for the redundant stimulus (black square) to PYRs (Hamm et al., 2018; in this case PYR1) and PVs (Chen et al., 2015). We propose that this synaptic depression on PYRs, inherited from multiple upstream synapses (e.g., onto thalamic relays) underlies SSA (Khatri et al., 2004; Yarden and Nelken, 2017), as depicted by the PYR1 trace. Indeed, SSA is present in thalamocortical inputs (Figure 3D; Khatri et al., 2004; Asari and Zador, 2009). PYR1 initially responds at a non-adapted level (Figure 3D; hashed line) but decreases response amplitude with each subsequent presentation. Similar adaptation has been observed in PYRs and PVs (Figure 3F; Reyes et al., 1998; Chen et al., 2015; Natan et al., 2017). In contrast, synaptic facilitation occurs on SSTs (Reyes et al., 1998), but since the inputs themselves may carry adaptation from further upstream, these effects lead to SSA which is present but less dramatic in SSTs (Figure 3G; Chen et al., 2015). Upstream input selective for the deviant stimulus (green square) remains unadapted, and presentations of the deviant stimulus induce PYR2 responses above the non-adapted level (Figure 3E; hashed line), i.e., classic “DD.”

This is due to at least three additional processes: (1) to open-state NDMA receptors, as pharmacological blockade of NMDA receptors diminishes MMN and oddball-drive responses/DD (Javitt et al., 1996; Chen et al., 2015). (2) DD also appears to depend on the action of SSTs, as inhibiting them selectively reduces DD (Natan et al., 2015; Hamm and Yuste, 2016). And (3) SSTs provide stronger inhibitory drive onto PVs than they do onto PYRs, and PVs impart stronger inhibition onto PYRs than SSTs do (Ma et al., 2010, 2012; Cottam et al., 2013; Natan et al., 2015; Safari et al., 2017). Thus (relatively) increased activity of SSTs throughout the paradigm leads to inhibition of PVs, which disinhibits all PYRs non-selectively, leading to the opening of NMDARs and supralinear responses (i.e., DD) in PYRs which are not adapted (i.e., PYR2 in this figure). Interestingly, a subset of SSTs is known to mediate disynaptic inhibition between PYRs (Silberberg and Markram, 2007). So alternatively, decreased activity of PYR1s, and thus reduced drive on mediating SSTs which inhibit PYR2s, may also contribute to the disinhibition (and opening of NMDARs) in PYR2s in the oddball paradigm before the presentation of the deviant. VIPs and other interneuron subtypes are an integral part of the neocortical circuit (Karnani et al., 2014), but as of now, little is known about their role in SSA or DD.

Notably, a number of the premises in Figure 3 come from studies of layer 2/3 neurons in primary sensory regions, so our confidence is strongest in this supragranular circuitry. Overall, like all models ours in Figure 3 is incomplete. For example, in another version of the oddball paradigm, DD has been reported in auditory cortex when a single auditory tone is used but the duration of the tone differs between the redundant and the deviant, known as “duration” mismatch (Schall et al., 2003; Colin et al., 2009; Peter et al., 2010; Schirmer et al., 2015). Duration MMN is also affected in SZ (Koshiyama et al., 2020). Although our model (Figure 3) is conceived with separate pitch or orientation-selective cortical ensembles, duration sensitive neurons have been identified in the auditory cortex (Beukes et al., 2009; Wang et al., 2016). Therefore, possibly, the dynamics among separate ensembles selective for the duration (or other non-identity stimulus features such as intensity, source localization, etc; Frey et al., 2015) may contribute to duration MMN and DD through similar intracortical circuit described in our model. Whether DD can exist in the absence of two separate PYR ensembles is unclear, although it could be apparent that it arises this way locally *via* feedback. For example, perhaps the interplay between duration selective neurons in a down-stream region (e.g., down-stream to A1) could effectively give rise to DD and send it back up-stream (e.g., as feedback to A1). Such an experiment constitutes an important test for our model.

Further, much of the SSA and DD work on which it is based, for instance, come from different sensory modalities (e.g., visual, auditory, and somatosensory), which may (Latimer et al., 2019) or may not (Kremlacek et al., 2016) exhibit distinct local circuitry for processing context. Further, distinct subpopulations exist within the SST-interneuron class which exhibit net inhibitory or net disinhibitory effects on PYRs (e.g., layer 4 × 94 cells; Muñoz et al., 2017). It remains possible that distinct subpopulations of interneurons, even within the same interneuron class (e.g., SSTs) differentially contribute to SSA and DD. Finally, it does not directly account for top-down influences nor information inherited from subcortical structures (Nelken and Ulanovsky, 2007; Stefanics et al., 2014, 2016; Carbajal and Malmierca, 2018). More rigorous studies across sensory systems are necessary to develop a cohesive and complete model of SSA and DD.

## Future Studies

Though incomplete, the proposed model may prove useful in guiding specific experiments in future work, especially in mice, where access to interneuron subpopulations is significant. First, a clearer picture of how SSTs, VIPs, PVs, and PYRs respond during oddball experiments in awake animals needs to be established. In particular, direct VIPs recordings in this paradigm have not been reported. Additional work should establish whether VIPs exhibit dynamics as hypothesized in Figure 3H, and/or SSA, DD, or some repetition facilitation (an inverse SSA).

Second, causal roles for PVs and VIPs have not been thoroughly examined in the context of DD (refer to Natan et al., 2015, 2017; regarding a role for PVs in auditory SSA Opto- or chemicogenetic experiments to target these populations while monitoring DD/SSA/MMN in the local cortex, like e.g., Hamm and Yuste (2016), are warranted.

Third, interactions between interneuron subtypes, namely that SSTs, PVs, and VIPs inhibit each other during specific parts of the oddball paradigm to effectively disinhibit subsets of PYRs, is key to this model. Imaging one population (e.g., PVs) while stimulating/inhibiting another (e.g., SSTs) and measuring gross LFP output would be a suitable test of these aspects of the model, and such an experiment is possible through the use of double transgenic strategies involving both Cre-and Flp- dependent gene expression in a complementary fashion (He et al., 2016).

Fourth, whether upstream inputs onto SSTs vs. PYRs are facilitated during this paradigm should be directly explored. This is a challenging experiment, but it is theoretically possible using 2P calcium imaging of axon boutons (Hamm et al., 2018), a combination of retrograde viral and cre-dependent expression (to express GCaMP in only upstream neurons synapsing on SSTs), and dual-color imaging (to colocalize boutons on SST dendrites).

Finally, a strong test of this model would be a demonstration that DD-like computations can be generated in isolated cortical circuits. *In vivo* recordings in the sensory cortex during an oddball paradigm followed by precise subsequent *ex vivo* recordings of the same neuronal populations in slices (Karnani et al., 2016) combined with patterned optogenetic stimulation (Carrillo-Reid et al., 2019) of two separate input populations (simulating the redundant and deviant preferring neurons) to generate and test adaptation and DD-like facilitation is possible, though, again, challenging. If SSA-like and DD-like processing are identified in such a setup, this would not only open the door for highly precise circuit-level investigations but could also revolutionize how MMN-deficits are understood.

## Conclusions and Clinical Significance

Recent results obtained from animals using the oddball and various control sequences demonstrates SSA and DD are not only separable, but likely arise due to different mechanisms. Our synthesis of this data and hypothesized model (Figure 3) suggests that DD in layer 2/3 of neocortex depends on the presence of adaptation in a subset of the local network and/or in afferents from the thalamus or layer 4, but this requires additional investigation. In both humans and animals, it appears DD is facilitated by and functionally related to the degree of SSA (Taaseh et al., 2011; Chien et al., 2019). Together these results highlight the complementary but distinct nature of SSA and DD. Furthermore, while the two processes have separable mechanisms, they also have mechanistic commonalities. For example, both are regulated by overall excitatory-inhibitory tone but different neuromodulators exert independent effects (Garrido et al., 2009). Future work is required to further elucidate the pathways and mechanisms required to generate adaptation and detection of deviation from expected patterns, especially when contexts become more complex than e.g., an “oddball paradigm.”

Both SSA and DD are co-represented in the human neurophysiological MMN response. While MMN is known to be altered relative to neurotypical controls in several neuropsychiatric diseases, such as SZ, autism spectrum disorders, and major depressive disorder among several others, many of these exhibit shared and distinct genetic risk (Brainstorm et al., 2018), neural pathophysiology (Mitelman, 2019), and symptoms of attentive or pre-attentive deficits that may lead to altered MMN for similar and distinct reasons. Animal research to understand the (likely) myriad neural circuit mechanisms underlying MMN will help in interpreting this biomarker in a neuropsychiatric setting (e.g., linking specific interneuron pathology to specific aspects of the MMN), but without differentiating the components of SSA and DD within the MMN measure during human experimentation, translational leaps will be difficult. Besides, such information would broaden our greater understanding of how the brain recognizes the change in its environment, a function with significant basic survival implications.

## Author Contributions

JR and JH discussed and contributed to the final manuscript.

## Conflict of Interest

The authors declare that the research was conducted in the absence of any commercial or financial relationships that could be construed as a potential conflict of interest.
